# Exopolysaccharide-associated protein sorting in environmental organisms: the PEP-CTERM/EpsH system. Application of a novel phylogenetic profiling heuristic

**DOI:** 10.1186/1741-7007-4-29

**Published:** 2006-08-24

**Authors:** Daniel H Haft, Ian T Paulsen, Naomi Ward, Jeremy D Selengut

**Affiliations:** 1 The Institute for Genomic Research, 9712 Medical Center Drive, Rockville MD 20850, USA

## Abstract

**Background:**

Protein translocation to the proper cellular destination may be guided by various classes of sorting signals recognizable in the primary sequence. Detection in some genomes, but not others, may reveal sorting system components by comparison of the phylogenetic profile of the class of sorting signal to that of various protein families.

**Results:**

We describe a short C-terminal homology domain, sporadically distributed in bacteria, with several key characteristics of protein sorting signals. The domain includes a near-invariant motif Pro-Glu-Pro (PEP). This possible recognition or processing site is followed by a predicted transmembrane helix and a cluster rich in basic amino acids. We designate this domain PEP-CTERM. It tends to occur multiple times in a genome if it occurs at all, with a median count of eight instances; *Verrucomicrobium spinosum *has sixty-five. PEP-CTERM-containing proteins generally contain an N-terminal signal peptide and exhibit high diversity and little homology to known proteins. All bacteria with PEP-CTERM have both an outer membrane and exopolysaccharide (EPS) production genes. By a simple heuristic for screening phylogenetic profiles in the absence of pre-formed protein families, we discovered that a homolog of the membrane protein EpsH (exopolysaccharide locus protein H) occurs in a species when PEP-CTERM domains are found. The EpsH family contains invariant residues consistent with a transpeptidase function. Most PEP-CTERM proteins are encoded by single-gene operons preceded by large intergenic regions. In the Proteobacteria, most of these upstream regions share a DNA sequence, a probable cis-regulatory site that contains a sigma-54 binding motif. The phylogenetic profile for this DNA sequence exactly matches that of three proteins: a sigma-54-interacting response regulator (PrsR), a transmembrane histidine kinase (PrsK), and a TPR protein (PrsT).

**Conclusion:**

These findings are consistent with the hypothesis that PEP-CTERM and EpsH form a protein export sorting system, analogous to the LPXTG/sortase system of Gram-positive bacteria, and correlated to EPS expression. It occurs preferentially in bacteria from sediments, soils, and biofilms. The novel method that led to these findings, partial phylogenetic profiling, requires neither global sequence clustering nor arbitrary similarity cutoffs and appears to be a rapid, effective alternative to other profiling methods.

## Background

Targeting signals in bacterial proteins often involve short, well-conserved motifs adjacent to predicted transmembrane helices. The lipoprotein signal sequence, the type IV pilin-like cleavage/methylation signal sequence, the twin-arginine transport (TAT) signal sequence, and the YSIRK-type signal sequence of some Firmicutes all represent classes of N-terminal signal sequence in which specific conserved residues are known or presumed [1-6] to interact with specific cellular machinery. The carboxyl-terminal LPXTG signal for targeting to the cell wall in Gram-positive bacteria and processing by sortase [7] is described as part of a larger sequence region with a transmembrane segment [8]. It appears that in some species multiple sortases act preferentially on different subclasses of LPXTG-like signals [9,10]. The processing by sortase includes proteolysis between the Thr and Gly residues of the LPXTG sequence [11] mediated by a catalytic triad composed of arginine, histidine and a cysteine nucleophile [12], followed by transfer of the C-terminal peptide to the cell wall precursor lipid II [13]. It is a reasonable hypothesis that other, to date undescribed, protein sorting systems exist in prokaryotes, particularly in Gram-negative species, which contain outer membranes.

Phylogenetic profiling is a methodology that was developed to determine the functional connections between protein homology families based on co-occurrence in multiple genomes [14]. The method depends on the assumption that genes with related functions will be retained together and, in the case of lateral gene transfer, transmitted together in order to preserve the biological process that they commonly serve. As initially construed, profiling requires a query profile (a list of genomes in which the "known" protein occurs), subject profiles for all other protein families, and a method for scoring the similarity between the query and subject profiles. One limitation of this methodology lies in the generation of the proper subject profiles. In the ideal scenario these would correspond to protein families, each with the property that all members are descended from a common ancestor and share a common function, where more distantly related proteins differ in function. Such protein families differ from ortholog families in two ways, namely that conserved protein function is a defining characteristic of the family and that lateral gene transfer may be part of its evolutionary history. We have suggested the term, 'equivalog'[15] for any such set of functionally conserved proteins, but note that equivalog families are built to reflect published characterizations and are not available for all proteins.

Clusters of genes (profiles) have been generated based on arbitrary BLAST similarity scores [14], graphs of bi-directional best (BLAST) hits [16,17], hierarchical clustering of BLAST scores [18], curated ortholog families [19], and PSI-BLAST-generated families [20]. In all cases, the parameters are set in an *ad hoc *manner based on the observation of acceptable behavior for known orthologous families. Difficulties necessarily arise from the circumstance of clusters that are too small (several related clusters have the same function) or too large (clusters include genes of heterogeneous function). This variable granularity in the similarity-to-function relationship among protein families appears to be inherent to the evolution of protein sequences. The global generation of clusters is often a computationally intense process, and may be made more so by the necessity of recalculating the clusters based on modified parameters for a particular application. In principle, correctly built protein families are highly preferable to mere sorted lists of pairwise matches for performing phylogenetic profiling, but there may be no way to know in advance how to set the granularity of the protein clustering method correctly to solve a particular biological problem.

We describe below a simple but powerful heuristic for scanning the set of all proteins from a test genome against a phylogenetic profile by examining their respective ordered lists of best BLAST hits from other genomes. An optimal cutoff is chosen separately for each hits list, at the point where the number of genomes encountered so far marked YES in the profile, vs the number marked NO, shows the greatest statistical significance according to the binomial distribution. This method may rapidly identify proteins of interest without any explicit construction of protein families. The method may be repeated for different test genomes in order to see if members of the same protein families consistently emerge as the top match to the profile, or amongst the top matches.

The heuristic we describe is not forced to evaluate the full phylogenetic profile. Rather, it performs an exploration of a section of the profile shown to be relevant by pairwise sequence similarity, that is, for those genomes where proteins score above the BLAST match self-optimizing cutoff score; we suggest 'partial phylogenetic profiling' as a name for this method. A phylogenetic profile across the set of complete genomes of a protein family, or of a genome property, may be imagined as a 'bar code'; one complete bar code can be compared to another, and the best correspondence found. The metaphor for our method is bar codes partially illuminated by a beam of light with an adjustable width; the width chosen is that which gives the most significant match of the illuminated section to the corresponding portion of the reference profile. We do not claim our method is better than other profiling techniques, but rather that it may work particularly well for certain classes of problem. When precomputed all-vs-all BLAST search results are available, our heuristic can run about one minute, and in our hands has produced meaningful results.

Hidden Markov Models representing the PEP-CTERM domain, EpsH and other relevant protein families described below have been deposited in the TIGRFAMs library [21] and have been combined to form a Genome Property (GenProp0326) [22] which is accessible through the Comprehensive Microbial Resource [23].

## Results and discussion

### PEP-CTERM domain proteins

We first encountered PEP-CTERM as a novel C-terminal homology domain among a set of predicted protein sequences of *Verrucomicrobium spinosum *(N. Ward, unpublished results), where all sequence regions with matches by Hidden Markov Models (HMMs) for previously defined protein families had been removed. We expanded this family by iteratively searching a set of all proteins from completed prokaryotic genomes with the current version of the HMM, aligning the set of matching sequences, building an HMM from the alignment, and starting again with a new sequence search. By this approach, we developed a model that detects over 300 sequences from 19 different genomes, out of 280 genomes searched. Our model has been deposited in the TIGRFAMs database [21] as TIGR02595. In 16 out of 19 genomes, the number of matches to the model was 4 or greater (Table 1).

Sequence similarity among some member proteins was encountered previously by Studholme, et al. [24] during a cataloguing of fifteen novel paralogous families with ten or more members in *Rhodopirellula baltica*. Their interpretation, reflected in model PF07589 in the Pfam database [25,26], is a 38 amino-acid region of unknown function whose length would require the sequence to continue past the stop codon for many instances of the PEP-CTERM region as we define it. It also continues upstream into regions of homology between homologous protein pairs in which one has, but the other lacks, the PEP-CTERM domain; comparing protein architecture in this way argues for interpretation of PEP-CTERM as module only about 24 amino acids in length. A subset of proteins that match TIGR02595, amounting to 22% of instances we detect, are recognized by the Pfam HMM PF07589. The model PF07589 does not find the set from *V. spinosum*.

**Figure 1:**
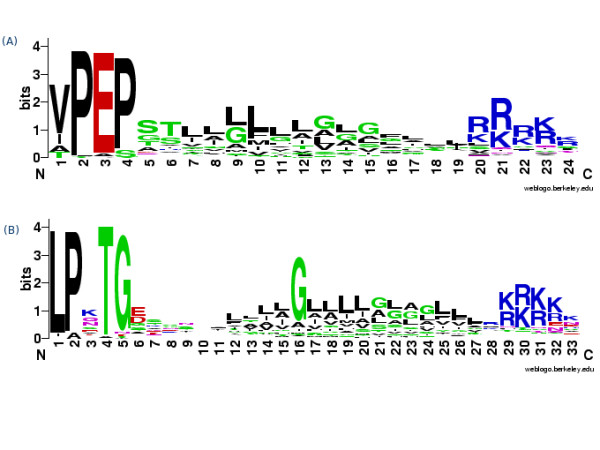
a) Consensus sequence LOGO [27] of the PEP-CTERM motif. PEP-CTERM consists of a highly conserved Proline-Glutamate-Proline triad followed by a hydrophobic putative transmembrane region and finally a positively charged segment. b) Consensus sequence LOGO of the LPXTG motif.

PEP-CTERM domains occur only in bacteria that possess an inner membrane, a periplasm, an outer membrane, and a complement of genes that suggests the synthesis of exopolysaccharide (see below). LPXTG domains occur almost exclusively in bacteria with a Gram-positive type cell envelope, that is, with a single cellular membrane outside of which is a cell wall. Figure 1 shows sequence logos [27] calculated from the seed alignments (columns with >50% gaps removed) for our models TIGR02595, for the PEP-CTERM domain, and TIGR01167, for the LPXTG domain. These logos show several features in common. Each shows a conserved motif, followed by a hydrophobic stretch likely to act as a transmembrane alpha helix, followed by a cluster of basic residues. Basic clusters adjacent to a transmembrane helix typically mark the cytosolic side of the bacterial inner membrane according to the "positive inside rule" [28,29]. Predictions of transmembrane helices and membrane topology of multiple PEP-CTERM proteins using MEMSAT3 [30] support this proposition, locating the conserved PEP motif precisely at the periplasm-membrane interface (data not shown).

We aligned all detected PEP-CTERM domains according to TIGR02595 and grouped related sequences by constructing a neighbor-joining tree in the alignment viewer Belvu according to their Scoredist sequence distances [31]. Inspection of the tree (data not shown) shows a clade of 9 PEP-CTERM domains from *Colwellia psychreythraea *34H all more closely related to each other than to any instance of the domain from other species. Other clades are similarly rich in just one or two species, including one of 47 sequences where 44 belong to *V. spinosum*, one of 20 where 17 belong to *Nitrospira multiformis *ATCC 25196 and the rest to *Thiobacillus denitrificans *ATCC 25259, and one of 39 where all belong to *Nostoc sp*. PCC 7120 or *Anabaena variabilis *ATCC 29413. This pattern suggests rapid paralogous family expansion of the PEP-CTERM domain in certain lineages.

The last two species mentioned above are a pair of Cyanobacteria so closely related that their RecA proteins, for example, show 98% sequence identity. Examining levels of sequence identity for full-length proteins rather than just the PEP-CTERM domain regions shows which sequence pairs are bidirectional best matches and putative orthologs. Among the 83 PEP-CTERM proteins of *Nostoc* and *Anabaena*, 27 pairs of putative orthologs were found, but also 29 proteins unique to one or the other. An HMM search against the translations into all six reading frames of the full genomic sequence showed that there was no fault in the identification of PEP-CTERM genes, as all genes had been properly predicted. The large number lacking apparent orthologs from one species to the other seems to point instead to frequent gene duplication and gene loss. Similar patterns are seen for other pairs of PEP-CTERM-positive species of the same genus. We conclude that the PEP-CTERM tail region marks some of the most rapidly evolving protein sets in their respective genomes.

**Figure 2:**
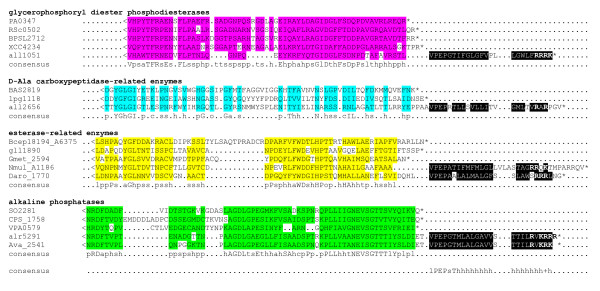
Sequence alignments of the C-terminal ends of enzyme families in which at least one member has an appended PEP-CTERM domain. (In the consensus sequences: a = aromatic, c = charged, h = hydrophobic, l = aliphatic, o = hydroxyl, p = polar, s = small, t = tiny, + = positive).

The PEP-CTERM sequences we detect are found near the extreme C-termini of their respective proteins. The twenty-four residue sequence regions described by the HMM end close to the extreme C-terminus of each protein, with 87% of proteins having fewer than ten additional residues; the median is three. The very few with dramatically longer tails may represent non-functional examples, as those with atypical locations within proteins tend to vary more in sequence than those examples in more typical locations (data not shown). An illustration of the PEP-CTERM region as a C-terminal domain appended to proteins whose homologs lack the domain is shown in Figure 2. These enzyme homologs are rare among PEP-CTERM-containing proteins. Typically PEP-CTERM proteins are observed to have no discernable homology to any other protein outside of the PEP-CTERM domain itself. Fewer than 10% match any protein family in Pfam. It was notable, however, that most PEP-CTERM containing proteins contained N-terminal signal peptide regions as predicted by SignalP 3.0 [32] with 95% confidence or higher. Due to the noted lack of homology we hypothesized that prediction of the proper start sites would have been problematic and many signal peptide sites may have been missed. Re-analysis of start site predictions for those proteins lacking signal peptides in completed genomes suggests that 97% of PEP-CTERM proteins also include a SignalP site (data not shown).

Excepting one three-member family of predicted integral membrane proteins, all members lack predicted transmembrane regions between the signal peptide region and the PEP-CTERM region. For each genome, we analyzed the amino-acid composition of all PEP-CTERM member proteins from the N-terminus to the beginning of the PEP-CTERM domain. Amino-acid frequencies for this protein cohort in each genome were compared to the average across all predicted proteins sequences in that genome (data not shown). The results consistently showed fewer basic residues, very low levels of Cys (0.4% +/- 0.2%), and substantial increases in the frequencies of Thr (from 5.4% +/- 0.4% to 8.8% +/- 1.4%), Ser (from 5.9% +/- 0.7% to 8.8% +/- 1.2%), and Asn (from 3.3% +/- 0.8% to 5.8% +/- 1.6%). We find it suggestive that these are the three typical amino acids for carbohydrate linkage in most known glycoproteins. A similar analysis for LPXTG proteins, which are known to be targeted to the cell surface, shows essentially the same pattern, suggesting a similar extracellular fate for PEP-CTERM proteins.

**Figure 3:**
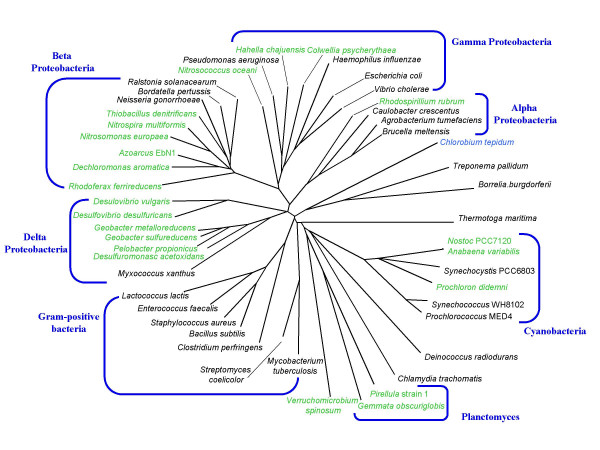
A phylogenetic tree based on 16S RNA of PEP-CTERM-containing and related organisms. Species with PEP-CTERM and an EpsH homolog are labeled in green. *Chlorobium tepidum*, which has only the PEP-CTERM domain, is shown in blue.

The phylogenetic distribution of PEP-CTERM-positive species is sporadic and somewhat sparse, being found broadly among Cyanobacteria, Planctomycetes, Verrumicrobiae, and the alpha, beta, gamma, and delta divisions of the Proteobacteria (Figure 3). All appear to be species of interest as environmental organisms and most if not all are associated with aquatic sediments and soils; none is reported as an animal pathogen. Three lineages that contain no example are the Archaea, the Firmicutes (low-GC Gram-positive), and the Actinomycetes (high-GC Gram-positive), which share the property of lacking both periplasm and outer membrane. As seen in Table 1, the PEP-CTERM domain count in a genome ranges from 1 to 65, with a median of 8 and a mean of over 14. Two species have two or fewer identified instances; the rest have four or more. The sporadic distribution of genomes with large numbers of PEP-CTERM regions, against a background of so many genomes with none at all, and so few genomes with just one or two, suggests to us that an abundance of PEP-CTERM modules marks the presence of some unknown biological system. The preponderance of signal peptide domains, the general lack of homology and the distinctive amino-acid distribution suggests to us that these proteins are destined for transport at least to the periplasm and possibly into or across the outer membrane. Finally, the highly conserved PEP motif and its position at the C-terminus suggest to us an interaction with some specific protein, analogous to the relationship in Gram-positive bacteria between LPXTG-like cell wall targeting sequences and sortases.

### Phylogenetic profiling identifies a correlated protein family: EpsH

We constructed a phylogenetic profile in which the value 1 (YES) is assigned to all genomes with more than two PEP-CTERM regions and 0 (NO) to all genomes with none. The two genomes with only one or two matching sequences are omitted from the profile entirely, for two reasons. First, a single sequence instance may score above the assigned cutoff score for our model simply because some error is intrinsic to any search method applied to very short sequence regions. Second, any biological system not required for viability may be lost, and single or low-scoring PEP-CTERM regions may be remnants of a non-functional system.

For fourteen genomes containing more than two PEP-CTERM proteins, we had pre-computed all-vs-all search results available to us through the CMR (Table 1) [23]. We tested each of the fourteen, in turn, as a reference genome for our method to find the proteins that best fit a phylogenetic profile (see methods, Table 2). For all fourteen genomes, the top candidate was a homolog of the *Methylobacillus sp*. 12S protein EpsH [33]. EpsH is a putative membrane protein based on its eight predicted transmembrane segments and is found within a large exopolysaccharide biosynthesis locus. We aligned these candidate sequences from complete genomes, constructed an HMM, performed a new search, and proceeded iteratively, resulting in TIGR02602. It identifies one or two members in every genome marked YES in the original profile and none in all genomes marked NO. This model also identifies a protein in *Rhodospirillum rubrum *ATCC 11170, a species omitted from the phylogenetic profile because we detect only one PEP-CTERM protein. All EpsH homologs identified by this model appear to have at least eight transmembrane helices as detected by TmHMM [34]. 90% of these proteins are found in the vicinity of exopolysaccharide biosynthesis associated genes. Despite this correlation, the disruption of EpsH in *Methylobacillus sp*. 12S proved not to affect production of methanolan, an exopolysaccharide whose synthesis depends instead on several neighboring genes [33]. According to Drummelsmith and Whitfield [35], Wzx and Wzy-related proteins with flippase activity show very low levels of sequence similarity but do show similarity in hydropathy profiles. Yoshida, *et al*. apply this principle to suggest that EpsH may be a flippase or a polymerase [33]. Its actual function was not determined.

We noted a single species, *Chlorobium tepidum*, in which the PEP-CTERM domain occurs twice, while no EpsH homolog protein is found. We note that *Desulfovibrio vulgaris *Hildenborough has a single plasmid with 152 reported genes [36], including a type III secretion system, a nitrogen fixation system, a CRISPR system [37], and a large tandem cluster of genes associated with exopolysaccharide biosynthesis including an EpsH homolog. Four of five PEP-CTERM proteins are found on the plasmid while a fifth is chromosomal. Loss of the plasmid would lead to a situation as in *Chlorobium tepidum*: no EpsH protein and only a small number (in this case one rather than two) of PEP-CTERM sequences. The nitrogen fixation plasmid is, in fact, easily cured in laboratory cultivation [36]; this finding supports the validity of assigning the YES state only to species with more than two PEP-CTERM instances in the PEP-CTERM phylogenetic profile. This threshold reduces noise from degenerate systems and/or false-positive detection of PEP-CTERM domains.

EpsH proteins do not themselves contain the PEP-CTERM domain. A number do, however contain an additional conserved C-terminal region which proves homologous to EpsI, encoded by the gene immediately downstream from EpsH in *Methylobacillus sp*. 12S. A model describing EpsI and homologous regions fused with or adjacent to other 8TM proteins has been built and deposited in the TIGRFAMs collection as TIGR02914. EpsI has no known function.

The epsH homolog gene nearly always is found among extracellular polysaccharide biosynthesis genes. This finding suggests to us a role of EpsH in processing PEP-CTERM proteins to an extracellular location. PEP-CTERM proteins could not both remain anchored to the inner membrane by the C-terminal transmembrane domain and basic cluster, and be exported like other components of exopolysaccharide. Cleavage of the anchoring sequence, analogous to the first step in the processing of LPXTG proteins by sortases, seems likely. LPXTG proteins are cleaved and become bound, covalently but transiently, to the active site cysteine of sortase [11]. Processing is completed when the LPXTG protein is attached covalently through an amide bond at its new carboxyl terminus to a peptidoglycan precursor, lipid II [13]. A histidine and an arginine, positioned in the folded protein near the active site cysteine [12], are both indispensable to function. The His, Cys, Arg catalytic triad residues are the only ones absolutely conserved in all sortases. The greatest local sequence conservation in a sortase multiple sequence alignment is centered on the active site Cys.

**Figure 4:**
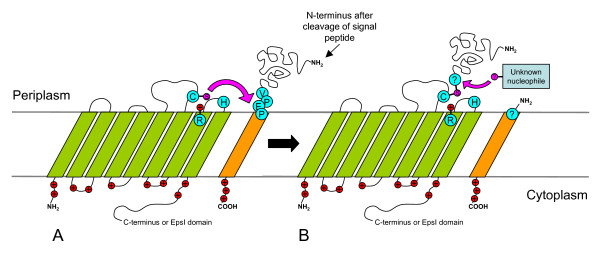
Proposed model of PEP-CTERM domain protein processing by EpsH. A) The PEP-CERM domain protein (orange) first is targeted to the inner membrane by its N-terminal signal peptide. Cleavage of the signal peptide by signal peptidase leaves the protein anchored by its C-terminal transmembrane helix. The PEP motif is predicted to lie at the membrane-periplasm interface where it is recognized by and binds to EpsH (green) adjacent to a catalytic triad on its periplasmic surface. Both the PEP-CTERM domain and EpsH are oriented in the membrane by their asymmetrical positive charge distribution (red). The cysteine sulfur nucleophile (purple) cleaves the bound PEP motif at an unspecified location. B) The covalently linked protein is subsequently transferred to an unknown periplasmic nucleophile (blue), likely destined to cross the outer membrane for incorporation into the exopolysaccharide layer.

The EpsH family is not homologous to sortases. EpsH proteins are highly hydrophobic with at least eight predicted transmembrane helices. In an alignment of all EpsH homologs, we found only six perfectly conserved residues, in three motifs with two conserved residues apiece. It has been shown from the analysis of multiple alignments and crystal structures that there is a high correlation between clusters of the most highly conserved residues in an alignment and functional sites; this relationship has been observed to be strongest for enzyme catalytic sites, rather than protein-protein and protein-DNA interaction sites, and to apply best when the overall conservation observed in the alignment is low [38]. This concept has been applied successfully to identify critical functional residues for the CAAX prenyl protease [39], a protein-sorting and modification enzyme useful for comparison because it, like EpsH, has multiple transmembrane segments. Low sequence identity is found for the EpsH family, with an overall percent identity of only 25 (particularly low considering its hydrophobic nature). The most highly conserved region in the whole alignment is centered on a Cys residue in the motif **Cys**-Xaa-Gly (bold type indicates residues found in the catalytic triad of sortases). Having its strongest local sequence conservation centered on an invariant Cys is a property the EpsH family shares with the sortases. Two other motifs with the remaining invariant residues follow downstream: Asn-Xaa-Xaa-**Arg **and **His**-Xaa-Xaa-Xaa-Gly. The membrane topology algorithm MEMSAT3 [30], used through the PSIPRED server [40], predicts that each residue of this putative catalytic triad (Cys, Arg, His) is located proximal to the outer surface of the membrane near the ends of three consecutive transmembrane helices (Figure 4). Notably, the outer surface of the inner membrane is the predicted location of the PEP motif, which is followed by a transmembrane helix and its cytoplasmic positively-charged anchor (Figure 1a). These observations suggest that the EpsH family protein may act in a manner mechanistically similar to sortase, with cleavage of the PEP-CTERM motif and transient attachment to an active site Cys, followed by further sorting and processing steps (Figure 4).

The strongly hydrophobic nature of EpsH family proteins seems more reminiscent of transporters than of proteases, but there is abundant precedent for protein sorting processes that depend on proteolytic cleavage and other processing by highly hydrophobic proteins. Proteins in eukaryotes with the CAAX motif, as found at the C terminus of Ras and other GTPases, are processed by isoprenylation, cleavage of the C-terminal tripeptide, and methylation of the isoprenylcysteine carboxyl group [39]. CAAX proteases such as CAAX prenyl protease 1 (SwissProt: P47154) and 2 (SwissProt: Q9Y256), active in this suite of modifications and essential for achieving correct localization of their targets, are highly hydrophobic integral membrane proteins. Pfam model PF02517 describes a domain shared by members of the CAAX prenyl protease family, although it is not clear which activity is associated with the domain described. In fact, we find four examples of fusion proteins with both EpsH-like and CAAX prenyl protease-like domains. By the principle of protein sequence Rosetta Stones [41], these fusions suggest a functional relationship between their respective domains and may further support a proposed role for EpsH homologs in the processing and sorting of PEP-CTERM proteins.

The perfect correlation to the phylogenetic profile of PEP-CTERM families makes details of the scoring heuristic rather unimportant for identifying EpsH, as any reasonable scoring scheme would agree. However, the ranked list of runners-up by our scoring method is informative. For eight of fourteen genomes tested against the PEP-CTERM profile, the list of all proteins with scores better than the arbitrarily selected cutoff of 6 (-logP, see Methods) is dominated by proteins encoded near on the chromosome to the EpsH member and/or tend to be implicated, by homology to better-characterized proteins, in exopolysaccharide biosynthesis (see, for instance, example 2 in Table 2). These include multiple examples of proteins identified by Pfam models PF00534 (glycosyl transferase, group 1), PF01522 (polysaccharide deacetylase), PF02706 (chain length determinant protein), and PF02397 (bacterial sugar transferase). Each of these other proteins, however, also shows strong sequence similarity to numbers of proteins found in genomes that lack PEP-CTERM modules. Only the EpsH family lacks detectable homologs in PEP-CTERM-negative species. Nevertheless, within the profile, members of these more weakly correlated gene families are almost invariably located in the vicinity of the EpsH homolog.

### Conserved DNA sequence upstream of PEP-CTERM domain proteins in proteobacteria

We noted that greater than 65% of PEP-CTERM proteins appear to belong to single-gene operons and to have large (greater than 200 nucleotides) regions of non-coding DNA upstream of the start codon. Following our investigation of PEP-CTERM gene start sites, it became clear that this was not an artifact of poor start site prediction. A review of these regions similarly failed to identify any reasonable open reading frames that might have been missed by gene calling.

**Figure 5:**
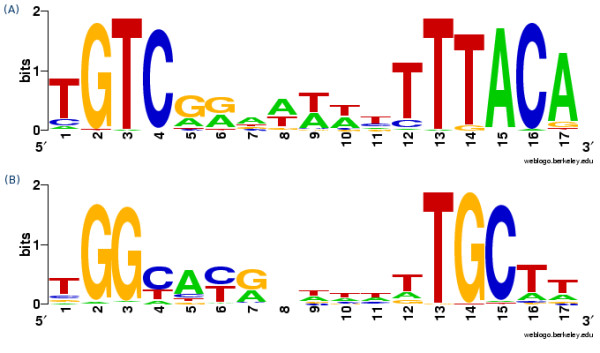
a) Consensus sequence LOGO [27] of motif 1 of the upstream region of PEP-CTERM genes in the Proteobacteria. Motif 1 is the predicted binding site for the PEP-CTERM regulatory system response regulator. b) Consensus sequence LOGO of motif. This motif includes the -24(GG)/-12(GC) pattern of RNA polymerase sigma-54 binding sites.

We searched for a conserved DNA sequence that could signify a regulatory site using a number of methods. The upstream regions of PEP-CTERM genes from genomes such as *A. variabilis*, *N. multiformis *and *C. psychrerythraea*, which have many such regions, were individually pooled for analysis. The program glam [42], which uses simulated annealing to find gapless local alignments in multiple sequences, produced an alignment in which a motif TTTACA appeared in a subset of regions, but only in the proteobacterial genomes. By exploring larger sequence regions, adding and removing sequences, and repeating rounds of search and realignment, we found the TTTACA sequence to be the 3'-end of a 17 nucleotide motif, motif 1. Motif 1 is sometimes repeated, as seen in the region before Rru_A3095, the only PEP-CTERM gene in *R. rubrum*. Motif 1, or the most 3' copy if repeated, is followed by a spacer region an average 62.8 nucleotides long (S.D. = 10.6, max = 92, min = 32), and then a different 17 nucleotide motif, motif 2. Sequence logos for these two motifs are shown in Figure 5.

For PEP-CTERM proteins preceded by such a DNA sequence region, the average distance from the end of motif 2 to the start codon is 71 nucleotides (S.D. = 38.5, max = -221, min = -23). Motif 2 conforms to the binding site consensus, -24(GG)/-12(GC), of sigma-54, the enhancer-dependent RNA polymerase sigma factor [43]. Regulation by the enhancer-dependent sigma factor allows for very tight regulation of expression, absolutely dependent on the enhancer binding protein, although at the cost of requiring longer stretches of intergenic DNA [44]. Motif 1 would therefore be the binding site for a cognate activator, a protein expected to bind both DNA and sigma-54.

We detected examples of this putative cis-regulatory sequence upstream of 99 of the 167 PEP-CTERM genes of proteobacteria. In some PEP-CTERM-positive genomes, we found a few additional sites upstream of other genes, but never within a protein-coding region. The few high-scoring matches from PEP-CTERM-negative genomes may all be false-positives, as most lie within protein-coding regions of those genomes and are unlikely to represent cis-regulatory elements.

Among the PEP-CTERM domain-containing species we discuss, it is only the members of the Proteobacteria that share these conserved DNA motifs. Among these proteobacterial, PEP-CTERM positive genomes, the observation of this motif is universal. This set of 13 Proteobacterial species comprises a new phylogenetic profile. The principle of using the phylogenetic profile of a DNA cis-regulatory site to identify a DNA-binding protein was demonstrated in the case of the NrdR-box associated with ribonucleotide reductase genes; leading to the identification of the NrdR-box binding protein [45]. We again applied our heuristic method for searching the gene list for matches to the profile, and again found that the set of candidate proteins we identified was independent of which genome was used as the starting point in the search. These identifications enabled us to construct seed alignments and HMMs to find all members of each family.

**Figure 6:**
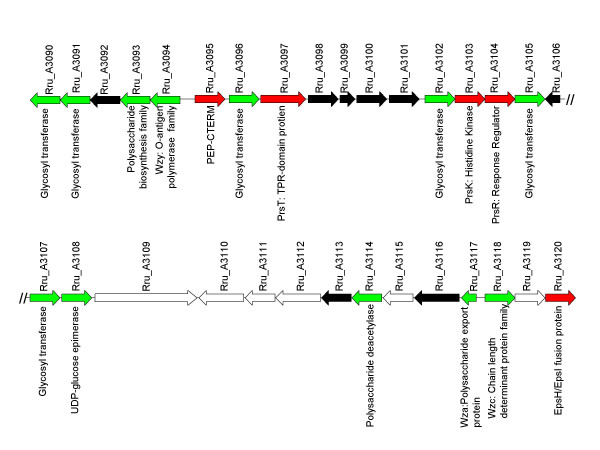
A 40 kilobase genomic region containing PEP-CTERM/EpsH system components and associated exopolysaccharide-related genes from *Rhodospirillum rubrum*. (Red = PEP-CTERM, Green = exopolysaccharide-related, Black = unknown functions, White = unrelated functions). Arrows show gene direction and relative size but are not to scale.

We found a set of three proteins that match the cis-regulatory site phylogenetic profile. The first is a predicted DNA-binding protein of the Fis family, with a sigma-54 interaction domain, as expected, plus a response regulator receiver domain. Its overall domain architecture identifies it as an enhancer-binding protein of the NtrC-like family [46]. The second is a transmembrane protein with a histidine kinase domain and an additional, uncharacterized domain. The third is a tetratricopeptide repeat (TPR) protein with a lipoprotein signal sequence. TPR domains participate in protein-protein interactions and may act as adaptors to guide protein covalent modifications, including a suggestive example in eukaryotes of an O-linked N-acetylglucosamine transferase that acts on a family of interacting proteins [47]. The histidine kinase and response regulator genes are usually tandem (12/13) and always at least close to each other. They are often in tandem with the TPR gene as well (4/13), sometimes near or adjacent to the EpsH gene (3/13), and usually near glycosyltransferases and other exopolysaccharide markers (12/13, Figure 6). We suggest the names (and associated gene symbols): PEP-CTERM regulatory system kinase (PrsK), response regulator (PrsR) and TPR domain protein (PrsT) for these genes (Table 1).

It is worth noting that methods of phylogenetic profiling based on homology clusters such as COGs, KEGG orthologs or PSI-BLAST would have made the discovery of these proteins far more difficult, if not impossible. PrsR, for instance, falls within COG2204 [16] as well as KEGG ortholog family KO2481 [19], each of which include sequences from a wide range of organisms outside the profile of interest. Similarly, use of PSI-BLAST draws over a thousand sequences into a cluster, the vast majority of which are response regulators with a similar architecture to, but distinct from PrsR.

### Association of the PEP-CTERM system with a particular biological niche

The association of PEP-CTERM system components with exopolysaccharide biosynthesis and transport genes must be considered against the fact that exopolysaccharide is expressed in a much wider group of organisms than PEP-CTERM. Exopolysaccharide is found expressed in organisms inhabiting a wide variety of biological niches including planktonic, biofilm-associated, terrestrial, sediment-associated, extremophilic and host-associated environments.

We scanned for keywords associated with the completed genomes in which we found PEP-CTERM at NCBI's Genome Projects site [48] and in the primary literature. This revealed a trend towards words (sediment, biofilm, soil, terrestrial) indicating association with or attachment to substrates, and away from those indicating free-living, extremophilic and host-associated environments. To further probe this trend we analyzed five environmental data sets, the survey of planktonic prokaryotes from the Sargasso Sea [49], the acidic mine drainage biofilm community from Iron Mountain, California [50], the Whale Fall Community of biofilms on a degrading whale carcass [50], the Yellowstone Hot Spring microbial mats (Heidelberg & Ward, unpublished data) and the surface farm soil sample from Waseca County, Minnesota [50]. Each nucleotide dataset was scanned (in each of the six possible translation frames) with the HMM for the PEP-CTERM domain (TIGR02595). This was compared to a similar analysis of all of the nucleotide data in the CMR representing a wide-ranging set of environments. The planktonic and acid-extremophilic environments were markedly lacking in PEP-CTERM genes, while those associated with biofilms and soils were relatively enriched (Table 3). In line with this observation, unfinished genome projects registered at the Genome Projects site were scanned for the keyword substrings "sediment," "biofilm," and "soil" and, where nucleotide data was available, were scanned for PEP-CTERM genes. A number of additional PEP-CTERM positive organisms were identified in this way (Table 4).

## Conclusion

We have now identified over 300 instances of a 24-amino-acid sequence region that we designate PEP-CTERM, which has three distinctive sequence features (Figure 1a). First, at the N-terminal end of the region, is a well-conserved segment where the tripeptide motif Pro-Glu-Pro (PEP) is nearly invariant. Next, a 14-residue strongly hydrophobic stretch forms a probable transmembrane (TM) helix. Last is a strongly basic five-residue region C-terminal to the TM helix likely to act as a cytoplasmic anchor.

The PEP-CTERM region is found, virtually without exception, near the protein C-terminus. Among the many sequenced genomes, instances are found only in bacteria that possess both inner and outer membranes. The majority of bacteria have no examples of PEP-CTERM, but those which do usually have many, averaging greater than 10. PEP-CTERM regions generally show much stronger sequence similarity within each species than between species, consistent with recent paralogous expansion in each lineage of PEP-CTERM as a modular domain. PEP-CTERM-family proteins have dramatically fewer recognizable homology domains on average, excepting the module itself, than do other proteins found in the same genome.

As the PEP-CTERM domain is probably too small to carry enzymatic activity or direct binding to a small molecule, the conservation of the nearly invariant Pro-Glu-Pro motif most likely reflects a physical interaction with another protein. Only the EpsH family has the appropriate profile to be the interacting partner. The hydrophobic nature of EpsH marks it as a probable membrane protein. Nearly all PEP-CTERM proteins have predicted signal peptides, and so would cross the inner membrane during translation concomitant with the cleavage of the signal peptide. At this point, we hypothesize the PEP-CTERM tail mediates an interaction with the EpsH family protein, which may entail further protein trafficking (Figure 4). A role in sorting for a C-terminal domain with a distinctive motif and a transmembrane segment is clearly possible, as seen for the LPXTG signal for cell wall targeting in Gram-positive bacteria. PEP-CTERM and LPXTG-mediated processes would differ, as the cell envelopes of Gram-negative and Gram-positive bacteria differ, however, the similarity of the observed triad of conserved residues in EpsH and sortase suggests that, like sortase, EpsH may be involved in the proteolysis (transpeptidation) of the associated proteins. LPXTG proteins do not then remain embedded in the plasma membrane, but are rather targeted to the cell wall. We propose, by analogy, those PEP-CTERM proteins, after proteolysis are targeted to, and possibly through the outer membrane. All PEP-CTERM-positive species have an outer membrane, just as all LPXTG-positive species have a cell wall. The physical properties of the two analogous domains are so similar that LPXTG domains show up among the top-scoring noise hits to PEP-CTERM. The small size of the domain suggests its purpose is to mark proteins that have it. The largely conserved gene neighborhood of the EpsH family proteins points to exopolysaccharide biosynthesis and export, yet mutational studies have shown that EpsH did not act on the exopolysaccharide itself. PEP-CTERM proteins appear to be disproportionally present in organisms living in biological niches characterized by substrates such as soils, sediments and biofilms where surface contact is likely to important. At this time, however, no experimental evidence exists concerning the cellular localization of any PEP-CTERM-containing protein.

The enhancer-dependent sigma factor binding site linked to proteobacterial PEP-CTERM genes is phylogenetically correlated to a transmembrane histidine kinase, which suggests an interaction with, and perhaps a contribution to, the external milieu of the cell. PEP-CTERM proteins are rich in the residues most likely to carry O-linked (Ser, Thr) and N-linked (Asn) carbohydrate. It seems likely to us that many PEP-CTERM term proteins are produced when exopolysaccharide is produced for biofilm formation and transported across the outer membrane where they add a proteinaceous component to the extracellular material. Experimental work will be required to determine when PEP-CTERM proteins are expressed, where they localize, whether they are processed at the C-terminus, and whether EpsH proteins are required for sorting and/or processing.

Should experimental findings verify the proposed proteolytic activity, we believe members of the EpsH family should be called "exosortase". This suggestion, for now, is based on the similar physical properties of PEP-CTERM and LPXTG transmembrane domains, the recurring association of EpsH genes with large exopolysaccharide biosynthesis loci, and the suggestion that the absolutely conserved residues Cys, Arg, and His found in the EpsH family are not merely fortuitously like the known catalytic triad His, Cys, and Arg (a different order) in the sortase family.

The proposed DNA motif and accessory proteins we have identified (PrsK, PrsR and PrsT) in the proteobacteria highlight the probable complex regulatory mechanisms that may be operating on the expression of PEP-CTERM proteins. The nature of the stimulus which triggers the activity of the histidine kinase PrsK remains obscure as the specificity domain appears to be entirely novel. Clearly, there are additional regulatory proteins and DNA motifs to be discovered in association with PEP-CTERM/EpsH systems, as many PEP-CTERM genes both within and outside of the proteobacteria have upstream non-coding regions not associated with the class described here.

## Methods

### Construction of hidden Markov models

#### Protein models

Preliminary multiple sequence alignments were constructed with Clustal W [51] or MUSCLE [52] and were trimmed and adjusted manually as needed. Hidden Markov models (HMMs) were constructed from multiple sequence alignments, and HMM searches were performed, using HMMER [53]. Previously described sequence domains present in proteins of interest were detected with Pfam [25,26] and TIGRFAMs [21]. Novel paralogous domains were detected by performing sequence similarity searching with BLAST [54] among protein sequence regions found to be negative for current TIGRFAMs and Pfam HMMs, followed by single linkage clustering. Sequences from clusters of interest were aligned by Clustal W and used to build HMMs. Preliminary search results were then used to expand the collection of sequences, realign, and improve the models. Criteria applied manually to judge candidate members of the PEP-CTERM family for inclusion in new rounds of model building included overall sequence similarity to other members, conserved position near the protein C-terminus, quality of the predicted transmembrane helix and other motifs, and consistency of the resulting multiple sequence alignment. Completed protein profile HMMs were deposited in the TIGRFAMs database [21].

#### DNA models

Significant similarity of DNA sequences in small, gapless alignments were detected by manual inspection of results from GLAM [55], followed by alignment of larger regions, with gaps, by Clustal W or MUSCLE. Several rounds of building a DNA profile HMM, searching intergenic regions, and producing new multiple sequence alignments from the search results were performed.

### Phylogenetic profiling; the partial phylogenetic profiling (PPP) methods

Phylogenetic profiles [14] were constructed from prokaryotic complete genomes in the Comprehensive Microbial Resource (CMR) [23] where the binary value 1 (YES) was assigned for genomes in which the reference feature was detected (more than 2 instances of the PEP-CTERM domain, or the presence of the conserved DNA motif), and 0 (NO) for all genomes in which it was not.

Using each profile-positive genome in turn, we scored every protein in that genome for its correlation between a phylogenetic profile and the list of related sequences in other genomes as measured by BLAST comparison. The heuristic operated as follows. The list of matching sequences from other genomes was sorted from the most significant match to the least. After the first occurrence of a species (i.e. multiple strains of the same species and/or multiple matches within the same genome), subsequent matches were ignored. Proceeding down the list of matched genomes, the relative counts of YES and NO states were scored at each point for the probability **P **that the current count **y **of YES genomes, or greater, could have been encountered by chance among the **N **genomes encountered so far, as calculated from the binomial distribution. The first term (see below) of the summation over all possible YES counts from **y **up to **N **dominates when YES genomes are relatively rare, but the full summation is performed. In the equation, **p **is the fraction of the species in the dataset, which are represented, in the positive (YES, 1) branch of the phylogenetic profile.

P = N!/(y!(N-y)!)·p^y^·(1-p)^(N-y)^

The best score at any point down the list does not necessarily occur when only YES states have been encountered. Consideration of the properties of the binomial distribution makes it obvious that, for a sparsely distributed property, finding, for instance, eleven genomes with ten marked YES and a single NO will be more significant than finding seven genomes all marked YES (Table 2). Although taxonomic filtering was used to minimize the bias introduced by the presence of multiple strains of the same species in the dataset, the resulting probabilities largely ignore the common ancestry of species with similar complements of proteins and should not be taken literally; calculated "probabilities" are used only for comparison among different proteins from the same genome. The match of any one protein from the reference genome to the phylogenetic profile becomes optimized for that protein by automatic selection of the most favorable BLAST E-value cutoff. The list of all proteins from the reference genome is then reported in order, starting with the most significant match to the phylogenetic profile. The top of the list can then be checked for findings of meaningful relationships. This heuristic avoids the need for precomputed protein families. While it depends on the choice of reference genome, it can be run separately for each genome in the profile. Our implementation uses pre-computed, comprehensive all-*versus*-all BLAST search results stored in the CMR [23]; the heuristic would work as well (but slower) with *de novo *searches.

To validate the phylogenetic profiling method, we constructed a phylogenetic profile in which all genomes with more than two matches to model TIGR01167, for the LPXTG cell wall anchor domain of Gram-positive bacteria, were assigned the value 1, while all with no matches were assigned 0. Note that this profile provides more information than a simple list of Gram-positive bacteria. For example, *Corynebacterium diptheriae *has many LPXTG domains, while *Corynebacterium glutamicum *has none. We tested four different reference genomes against this profile: *C. diptheriae *NCTC 13129, *Bacillus cereus *ATCC 14579, *Listeria monocytogenes *EGD-e, and *Streptococcus pneumoniae *R6. In all four cases, the top match identified to the profile was a member of the sortase family. The sortase SrtA is known to recognize LPXTG sequences and catalyze a transpeptidation that attaches the target protein to the cell wall [11]. This method, therefore, could have deduced the connection between LPXTG sequences and sortases had the relationship not already been known. Similarly, we constructed profiles for the presence of the twin-arginine transport (TAT) signal sequence (TIGR01409) and the type IV pilin-like N-terminal cleavage and methylation signal (TIGR02532). In each case, critical elements of the associated handling systems were readily identified (data not shown).

## Authors' contributions

DH and JS constructed the multiple sequence alignments, hidden Markov models, and PEP-CTERM genome property definition. JS and DH conceived the partial phylogenetic profile method, and JS wrote the computer program. NW led the *Verrucomicrobium spinosum *sequencing effort. JS, DH, and IP analyzed the cis-regulatory site. DH performed the sequence-function analysis of the EpsH protein family. IP performed the phylogenetic tree analyses. DH, JS, NW, and IP interpreted the results. DH and JS wrote the paper. JS, DH, NW, and IP revised the paper.

## Figures and Tables

**Table 1:** PEP-CTERM/EpsH system genes identified in completed genomes

**Species**	**PEP-CTERM **TIGR02595	**EpsH **TIGR02602	**EpsI **TIGR02914	**PrsK **TIGR2916	**PrsR **TIGR2915	**PrsT **TIGR02917
*Verrucomicrobium spinosum *DSM 4136	65	ORF00124 ORF01727	-	-	-	-
Nostoc sp. PCC 7120^a^	42	all0497	-	-	-	-
*Nitrospira multiformis *ATCC 25196^a^	39	Nmul_A0246 Nmul_A2521	Nmul_A2521	Nmul_A2672	Nmul_A2671	Nmul_A0376
*Anabaena variabilis *ATCC 29413^a^	36	Ava_2911	-	-	-	-
*Hahella chejuensis *KCTC 2396^a^	27	HCH_02741 HCH_03425	HCH_03424	HCH_02749	HCH_02748	HCH_02747
*Thiobacillus denitrificans *ATCC 25259^a^	21	Tbd_0290 Tbd_1792	Tbd_0290	Tbd_0198	Tbd_0197	Tbd_0195 Tbd_0196
*Colwellia psychrerythraea *34H	19	CPS_4990	-	CPS_5031	CPS_5030	CPS_0309 CPS_0310 CPS_0528 CPS_0781
*Dechloromonas aromatica *RCB^a^	16	Daro_2406	Daro_2406	Daro_2437	Daro_2438	Daro_1943 Daro_2439
*Rhodopirellula baltica *SH 1^a^	12	RB2461	-	-	-	-
*Geobacter metallireduscens *GS-15	10	Gmet_1503 Gmet_2003	Gmet_1503 Gmet_2002	Gmet_1993	Gmet_1992	Gmet_2032
*Nitrosomonas europaea *ATCC 19718^a^	8	NE1797	NE1797	NE0377	NE1124	NE1444
*Nitrosococcus oceani *ATCC 19707^a^	6	Noc_1778	Noc_1778	Noc_1998	Noc_1997	Noc_1987
*Geobacter sulfurreducens *PCA^a^	5	GSU1979	GSU1978	GSU1941	GSU1940	GSU1987
*Desulfovibrio vulgaris Hildenborough*^a^	5	DVU_A0055	DVU_A0056	DVU_A0065	DVU_A0057	DVU_A0036
*Desulfovibrio desulfuricans *G20	5	Dde_0847	Dde_0848	Dde_0849	Dde_0850	Dde_0828
Azoarcus sp. EbN1^a^	4	ebA4261	ebA4261	ebA38	ebA37	ebA36
*Rhodospirillum rubrum *ATCC 11170	1*	Rru_A3120	Rru_A3120	Rru_A3103	Rru_A3104	Rru_A3097
*Chlorobium tepidum *TLS	2*	-	-	-	-	-

^a ^Data from these genomes was used in the phylogenetic profiling studies that identified EpsH.

**Table 2:** Representative results from the partial phylogenetic profiling analysis of the predicted proteins of Nitrosomonas europaea ATCC 19718. The PEP-CTERM profile consisted of 12 positive (YES, 1) and 190 negative (NO, 0) species.

**BLAST E-value**	**Top hit locus**	**Hit genome**	**PEP-CTERM Profile**	**partial profile score -log(P)**
Best hit: NE1797 – EpsH				

2.1 e-142	Nmul_A2521	*Nitrospira multiformis*	1	**|||| 1.2**
1.6 e-112	Tbd_0290	*Thiobacillus denitrificans*	1	**|||||| 2.4**
3.0 e-102	Noc_1978	*Nitrosococcus oceani*	1	**||||||||| 3.7**
9.2 e-92	Daro_2406	*Dechloromonas aromatica*	1	**|||||||||||| 4.9**
2.7 e-78	ebA4261	Azoarcus sp. EbN1	1	**||||||||||||||| 6.1**
1.1 e-38	HCH_02741	*Hahella chejuensis*	1	**|||||||||||||||||| 7.4**
2.3 e-33	GSU_1979	*Geobacter sulfurreducens*	1	**||||||||||||||||||||| 8.6**
2.7 e-24	DVU_A0055	*Desulfovibrio vulgaris*	1	**|||||||||||||||||||||||| 9.8**
1.7 e-06	all5246	Nostoc sp. PCC 7120	1	**||||||||||||||||||||||||||| 11.0**
2.9 e-06	Ava_2911	*Anabaena variabilis*	1	**||||||||||||||||||||||||||||||| 12.3**
1.3 e-03	RB2461	*Rhodopirellula baltica*	1	**|||||||||||||||||||||||||||||||||| 13.5 ◀**
1.9 e-02	PSHAb0228	*Pseuroalteromonas haloplanktis*	0	**||||||||||||||||||||||||||||||| 12.4**
3.1 e-02	Plut_1390	*Pelodictyon luteolum*	0	**||||||||||||||||||||||||||||| 11.6**
8.0 e-02	AF_1831	*Archaeoglobus fulgidus*	0	**||||||||||||||||||||||||||| 11.0**

15^th ^best hit: NE2015 – possible CapK protein				

3.3 e-80	Daro_2411	* Dechloromonas aromatica *	1	**|||| 1.2**
1.3 e-76	Noc_1971	*Nitrosococcus oceani*	1	**|||||| 2.4**
1.9 e-75	Nmul_A0291	*Nitrospira multiformis*	1	**||||||||| 3.7**
2.4 e-54	ebA4317	Azoarcus sp. EbN1	1	**|||||||||||| 4.9**
8.4 e-43	Tbd_0295	*Thiobacillus denitrificans*	1	**||||||||||||||| 6.1**
1.0 e-28	MA1063	*Methanosarcina acetivorans*	0	**|||||||||||||| 5.4**
4.2 e-26	GSU1950	*Geobacter sulfurreducens*	1	**|||||||||||||||| 6.5 ◀**
2.0 e-20	NT02CA1188	*Chloroflexus aurantiacus*	0	**||||||||||||||| 6.0**
9.2 e-18	cbdbA894	Dehalococcoides sp. CBDB1	0	**|||||||||||||| 5.5**
1.3 e-16	VC0924	*Vibrio cholerae *El Tor	0	**||||||||||||| 5.1**
1.4 e-16	DVU_3253	*Desulfovibrio vulgaris*	1	**|||||||||||||||| 6.2**
5.1 e-16	lpl1943	*Legionella pneumophila*	0	**|||||||||||||| 5.8**
2.5 e-15	DET_0940	*Dehalococcoides ethenogenes*	0	**|||||||||||||| 5.5**
2.5 e-15	PSPTO_2429	*Pseudomonas syringae*	0	**||||||||||||| 5.2**

34^th ^best hit: NE1609 – type II secretion system protein E				

7.9e-221	Nmul_A2588	*Nitrospira multiformis*	1	**|||| 1.2**
2.5 e-170	Tbd_1295	*Thiobacillus denitrificans*	1	**|||||| 2.4**
2.0 e-160	Pfl_1982	*Pseudomonas fluorescens*	0	**||||| 2.0**
6.1 e-159	PP3483	*Pseudomonas putida*	0	**|||| 1.7**
5.4 e-158	Daro_3223	*Dechloromonas aromatica*	1	**||||||| 2.7**
7.4 e-154	ebA1226	Azoarcus sp. EbN1	1	**|||||||||| 3.8**
4.3 e-149	RSp0148	*Ralstonia solanacearum*	0	**|||||||| 3.4**
3.1 e-132	GSU1783	*Geobacter sulfurreducens*	1	**||||||||||| 4.4**
9.6 e-129	Pcar_0131	*Pelobacter carbinoicus*	0	**|||||||||| 4.1**
1.2 e-91	HCH_03937	*Hahella chejuensis*	1	**||||||||||||| 5.1 ◀**
1.1 e-90	TTE1262	*Thermoanaerobacter tengcongensis*	0	**|||||||||||| 4.8**
1.1 e-88	SO0167	*Shewanella oneidensis*	0	**||||||||||| 4.5**
1.4 e-88	Moth_1554	*Moorella thermoacetica*	0	**||||||||||| 4.3**

**Table 3:** PEP-CTERM domain abundance in environmental metagenome datasets

**ID**	**Environmental source**	**Size (MB)**	**# PEP-CTERM**	**Relative abundance^a^**
13694	Sargasso sea: planktonic (filtered) marine	670	2	0.01
13696	Iron Mountain, CA: acid mine drainage biofilm	10	1	0.4
	Yellowstone hot spring microbial mats	168	150	4
13700	Whale Fall Community: microbial mat & rib bone	9	18	8
13699	Waseca County, MN: clay loam surface farm soil	12	84	29

^a ^Data was normalized to results from the Comprehensive Microbial Resource which showed 256 hits from a database size of 1048 MB of DNA.

**Table 4:** PEP-CTERM domain abundance in incomplete genomes associated with sediments and biofilms

**ID**	**Species**	**Phylum**	**Niche**	**# PEP-CTERM**
15753	*Acidophilum cryptum *JF-5	α-proteobacteria	coal mine lake sediment	14
13418	*Polaromonas naphthalenivorans *CJ2	β-proteobacteria	contam. freshwater sed.	3
13908	*Rhodoferax ferrireduscens *DSM 15236	β-proteobacteria	aquifer sediment	14
12551	*Desulfuromonas acetoxidans *DSM 684	δ-proteobacteria	anoxic marine sediment	8
13384	*Pelobacter propionicus *DSM 2379	δ-proteobacteria	anaerobic sediments	9
13454	*Pseudoalteromonas atlantica *T6C	γ-proteobacteria	marine biofilm	13

## References

[B1] Juncker AS, Willenbrock H, Von Heijne G, Brunak S, Nielsen H, Krogh A (2003). Prediction of lipoprotein signal peptides in Gram-negative bacteria. Protein Sci.

[B2] Pugsley AP (1993). Processing and methylation of PuIG, a pilin-like component of the general secretory pathway of *Klebsiella oxytoca*. Mol Microbiol.

[B3] Tettelin H, Nelson KE, Paulsen IT, Eisen JA, Read TD, Peterson S, Heidelberg J, DeBoy RT, Haft DH, Dodson RJ (2001). Complete genome sequence of a virulent isolate of *Streptococcus pneumoniae*. Science.

[B4] Bae T, Schneewind O (2003). The YSIRK-G/S motif of staphylococcal protein A and its role in efficiency of signal peptide processing. J Bacteriol.

[B5] Santini CL, Ize B, Chanal A, Muller M, Giordano G, Wu LF (1998). A novel sec-independent periplasmic protein translocation pathway in *Escherichia coli*. EMBO J.

[B6] Berks BC (1996). A common export pathway for proteins binding complex redox cofactors?. Mol Microbiol.

[B7] Lee SG, Pancholi V, Fischetti VA (2002). Characterization of a unique glycosylated anchor endopeptidase that cleaves the LPXTG sequence motif of cell surface proteins of Gram-positive bacteria. J Biol Chem.

[B8] Navarre WW, Schneewind O (1999). Surface proteins of gram-positive bacteria and mechanisms of their targeting to the cell wall envelope. Microbiol Mol Biol Rev.

[B9] Marraffini LA, Schneewind O (2005). Anchor structure of staphylococcal surface proteins. V. Anchor structure of the sortase B substrate IsdC. J Biol Chem.

[B10] Comfort D, Clubb RT (2004). A comparative genome analysis identifies distinct sorting pathways in gram-positive bacteria. Infect Immun.

[B11] Ton-That H, Mazmanian SK, Faull KF, Schneewind O (2000). Anchoring of surface proteins to the cell wall of *Staphylococcus aureus*. Sortase catalyzed *in vitro *transpeptidation reaction using LPXTG peptide and NH(2)-Gly(3) substrates. J Biol Chem.

[B12] Zong Y, Bice TW, Ton-That H, Schneewind O, Narayana SV (2004). Crystal structures of *Staphylococcus aureus *sortase A and its substrate complex. J Biol Chem.

[B13] Ruzin A, Severin A, Ritacco F, Tabei K, Singh G, Bradford PA, Siegel MM, Projan SJ, Shlaes DM (2002). Further evidence that a cell wall precursor [C(55)-MurNAc-(peptide)-GlcNAc] serves as an acceptor in a sorting reaction. J Bacteriol.

[B14] Pellegrini M, Marcotte EM, Thompson MJ, Eisenberg D, Yeates TO (1999). Assigning protein functions by comparative genome analysis: protein phylogenetic profiles. Proc Natl Acad Sci USA.

[B15] Haft DH, Loftus BJ, Richardson DL, Yang F, Eisen JA, Paulsen IT, White O (2001). TIGRFAMs: a protein family resource for the functional identification of proteins. Nucleic Acids Res.

[B16] Tatusov RL, Fedorova ND, Jackson JD, Jacobs AR, Kiryutin B, Koonin EV, Krylov DM, Mazumder R, Mekhedov SL, Nikolskaya AN (2003). The COG database: an updated version includes eukaryotes. BMC Bioinformatics.

[B17] Mikkelsen TS, Galagan JE, Mesirov JP (2005). Improving genome annotations using phylogenetic profile anomaly detection. Bioinformatics.

[B18] Uchiyama I (2006). Hierarchical clustering algorithm for comprehensive orthologous-domain classification in multiple genomes. Nucleic Acids Res.

[B19] Ogata H, Goto S, Sato K, Fujibuchi W, Bono H, Kanehisa M (1999). KEGG: Kyoto Encyclopedia of Genes and Genomes. Nucleic Acids Res.

[B20] Wu H, Su Z, Mao F, Olman V, Xu Y (2005). Prediction of functional modules based on comparative genome analysis and gene ontology application. Nucleic Acids Res.

[B21] Haft DH, Selengut JD, White O (2003). The TIGRFAMs database of protein families. Nucleic Acids Res.

[B22] Haft DH, Selengut JD, Brinkac LM, Zafar N, White O (2005). Genome Properties: a system for the investigation of prokaryotic genetic content for microbiology, genome annotation and comparative genomics. Bioinformatics.

[B23] Peterson JD, Umayam LA, Dickinson T, Hickey EK, White O (2001). The Comprehensive Microbial Resource. Nucleic Acids Res.

[B24] Studholme DJ, Fuerst JA, Bateman A (2004). Novel protein domains and motifs in the marine planctomycete *Rhodopirellula baltica*. FEMS Microbiol Lett.

[B25] Bateman A, Coin L, Durbin R, Finn RD, Hollich V, Griffiths-Jones S, Khanna A, Marshall M, Moxon S, Sonnhammer EL, Studholme DJ, Yeats C, Eddy SR (2004). The Pfam protein families database. Nucleic Acids Res.

[B26] Finn RD, Mistry J, Schuster-Bockler B, Griffiths-Jones S, Hollich V, Lassmann T, Moxon S, Marshall M, Khanna A, Durbin R, Eddy SR, Sonnhammer EL, Bateman A (2006). Pfam: clans, web tools and services. Nucleic Acids Res.

[B27] Crooks GE, Hon G, Chandonia JM, Brenner SE (2004). WebLogo: a sequence logo generator. Genome Res.

[B28] von Heijne G (1992). Membrane protein structure prediction. Hydrophobicity analysis and the positive-inside rule. J Mol Biol.

[B29] Andersson H, von Heijne G (1994). Membrane protein topology: effects of delta mu H+ on the translocation of charged residues explain the 'positive inside' rule. EMBO J.

[B30] Jones DT, Taylor WR, Thornton JM (1994). A model recognition approach to the prediction of all-helical membrane protein structure and topology. Biochemistry.

[B31] Sonnhammer EL, Hollich V (2005). Scoredist: a simple and robust protein sequence distance estimator. BMC Bioinformatics.

[B32] Bendtsen JD, Nielsen H, von Heijne G, Brunak S (2004). Improved prediction of signal peptides: SignalP 3.0. J Mol Biol.

[B33] Yoshida T, Ayabe Y, Yasunaga M, Usami Y, Habe H, Nojiri H, Omori T (2003). Genes involved in the synthesis of the exopolysaccharide methanolan by the obligate methylotroph *Methylobacillus *sp strain 12S. Microbiology.

[B34] Krogh A, Larsson B, von Heijne G, Sonnhammer EL (2001). Predicting transmembrane protein topology with a hidden Markov model: application to complete genomes. J Mol Biol.

[B35] Drummelsmith J, Whitfield C (1999). Gene products required for surface expression of the capsular form of the group 1 K antigen in *Escherichia coli *(O9a:K30). Mol Microbiol.

[B36] Heidelberg JF, Seshadri R, Haveman SA, Hemme CL, Paulsen IT, Kolonay JF, Eisen JA, Ward N, Methe B, Brinkac LM (2004). The genome sequence of the anaerobic, sulfate-reducing bacterium *Desulfovibrio vulgaris Hildenborough*. Nat Biotechnol.

[B37] Haft DH, Selengut J, Mongodin EF, Nelson KE (2005). A guild of 45 CRISPR-associated (Cas) protein families and multiple CRISPR/Cas subtypes exist in prokaryotic genomes. PLoS Comput Biol.

[B38] Panchenko AR, Kondrashov F, Bryant S (2004). Prediction of functional sites by analysis of sequence and structure conservation. Protein Sci.

[B39] Pei J, Grishin NV (2001). Type II CAAX prenyl endopeptidases belong to a novel superfamily of putative membrane-bound metalloproteases. Trends Biochem Sci.

[B40] McGuffin LJ, Bryson K, Jones DT (2000). The PSIPRED protein structure prediction server. Bioinformatics.

[B41] Marcotte EM, Pellegrini M, Ng HL, Rice DW, Yeates TO, Eisenberg D (1999). Detecting protein function and protein-protein interactions from genome sequences. Science.

[B42] Tharakaraman K, Marino-Ramirez L, Sheetlin S, Landsman D, Spouge JL (2005). Alignments anchored on genomic landmarks can aid in the identification of regulatory elements. Bioinformatics.

[B43] Thony B, Hennecke H (1989). The -24/-12 promoter comes of age. FEMS Microbiol Rev.

[B44] Buck M, Gallegos MT, Studholme DJ, Guo Y, Gralla JD (2000). The bacterial enhancer-dependent sigma(54) (sigma(N)) transcription factor. J Bacteriol.

[B45] Rodionov DA, Gelfand MS (2005). Identification of a bacterial regulatory system for ribonucleotide reductases by phylogenetic profiling. Trends Genet.

[B46] Studholme DJ, Dixon R (2003). Domain architectures of sigma54-dependent transcriptional activators. J Bacteriol.

[B47] Iyer SP, Hart GW (2003). Roles of the tetratricopeptide repeat domain in O-GlcNAc transferase targeting and protein substrate specificity. J Biol Chem.

[B48] Entrez Genome Project. http://www.ncbi.nlm.nih.gov/entrez/query.fcgi?db=genomeprj.

[B49] Venter JC, Remington K, Heidelberg JF, Halpern AL, Rusch D, Eisen JA, Wu D, Paulsen I, Nelson KE, Nelson W (2004). Environmental genome shotgun sequencing of the Sargasso Sea. Science.

[B50] Tringe SG, von Mering C, Kobayashi A, Salamov AA, Chen K, Chang HW, Podar M, Short JM, Mathur EJ, Detter JC (2005). Comparative metagenomics of microbial communities. Science.

[B51] Thompson JD, Higgins DG, Gibson TJ (1994). CLUSTAL W: improving the sensitivity of progressive multiple sequence alignment through sequence weighting, position-specific gap penalties and weight matrix choice. Nucleic Acids Res.

[B52] Edgar RC (2004). MUSCLE: multiple sequence alignment with high accuracy and high throughput. Nucleic Acids Res.

[B53] HMMER: sequence analysis using profile hidden Markov models. http://hmmer.wustl.edu.

[B54] Altschul SF, Madden TL, Schaffer AA, Zhang J, Zhang Z, Miller W, Lipman DJ (1997). Gapped BLAST and PSI-BLAST: a new generation of protein database search programs. Nucleic Acids Res.

[B55] Frith MC, Hansen U, Spouge JL, Weng Z (2004). Finding functional sequence elements by multiple local alignment. Nucleic Acids Res.

